# Honey Bee Workers That Are Pollen Stressed as Larvae Become Poor Foragers and Waggle Dancers as Adults

**DOI:** 10.1371/journal.pone.0121731

**Published:** 2015-04-08

**Authors:** Hailey N. Scofield, Heather R. Mattila

**Affiliations:** Department of Biological Sciences, Wellesley College, Wellesley, Massachusetts, United States of America; University of North Carolina, Greensboro, UNITED STATES

## Abstract

The negative effects on adult behavior of juvenile undernourishment are well documented in vertebrates, but relatively poorly understood in invertebrates. We examined the effects of larval nutritional stress on the foraging and recruitment behavior of an economically important model invertebrate, the honey bee (*Apis mellifera*). Pollen, which supplies essential nutrients to developing workers, can become limited in colonies because of seasonal dearths, loss of foraging habitat, or intensive management. However, the functional consequences of being reared by pollen-stressed nestmates remain unclear, despite growing concern that poor nutrition interacts with other stressors to exacerbate colony decline. We manipulated nurse bees’ access to pollen and then assessed differences in weight, longevity, foraging activity, and waggle-dance behavior of the workers that they reared (who were co-fostered as adults). Pollen stress during larval development had far-reaching physical and behavioral effects on adult workers. Workers reared in pollen-stressed colonies were lighter and shorter lived than nestmates reared with adequate access to pollen. Proportionally fewer stressed workers were observed foraging and those who did forage started foraging sooner, foraged for fewer days, and were more likely to die after only a single day of foraging. Pollen-stressed workers were also less likely to waggle dance than their unstressed counterparts and, if they danced, the information they conveyed about the location of food was less precise. These performance deficits may escalate if long-term pollen limitation prevents stressed foragers from providing sufficiently for developing workers. Furthermore, the effects of brief pollen shortages reported here mirror the effects of other environmental stressors that limit worker access to nutrients, suggesting the likelihood of their synergistic interaction. Honey bees often experience the level of stress that we created, thus our findings underscore the importance of adequate nutrition for supporting worker performance and their potential contribution to colony productivity and quality pollination services.

## Introduction

The negative impact of developmental nutritional stress on adult function has been documented across a wide range of vertebrates (reviewed by [1–7]). The functional consequences of early food stress are diverse, including impaired learning and song production in adult songbirds [8], poor response of frogs to pond drying [9], and late-onset metabolic and neurodevelopmental disorders in humans [10–13]. In contrast, our understanding of the impact of developmental undernutrition on adult performance in invertebrates is comparatively slim. Studies with model insects show that inadequate access to food during larval development may slow “rate of living” [14] by decreasing growth and onset of reproduction as an adaptive strategy for withstanding food scarcity (e.g., fruit flies [15,16]; ladybird beetles [17]; mosquitoes [18]), which can result in lower adult body weight and impose reproductive costs on individuals [19–22]. Few insect studies have examined the effects of larval food stress on non-reproductive adult behavior, although it has been shown to decrease flight metabolism and territory defense in butterflies [23,24] and alter exploratory foraging, learning ability, and memory in adult fruit flies [25,26].

The goal of this study is to evaluate the consequences of larval food stress for adults of another important model invertebrate—the honey bee (*Apis mellifera* L.). Honey bees’ strategy for dealing with food stress is complicated by eusociality, where “rate of living” adjustments are challenged by the enormous energy and labor demands of collective brood rearing, foraging, and nest maintenance. The need to understand the potential effects of nutritional stress on honey bees has become urgent in recent years. Honey bees pollinate almost half of the crops that are cultivated worldwide for use by humans [27] and are key contributors to the estimated €153 billion (~$211 billion USD) total annual value of insect pollination [28]. However, honey bees’ role in global food production has been threatened in recent years by unusually high mortality of managed colonies [29–38] coupled with declining colony numbers during the preceding decades [36,39]. Consensus is growing that the effects and interactions of a multi-factorial set of stressors, including parasites, pathogens, pesticides, low genetic diversity, and poor nutrition, is causing or exacerbating these losses [39–41]. Nutritional stress is a particular concern because it can act synergistically with other environmental stressors [42,43] and it is presumed to worsen in areas with shrinking foraging habitat, where colony losses are the greatest [44].

The chief source of nutritional stress in colonies is inadequate access to pollen, which provides the essential protein, lipids, vitamins, and minerals that are required for larval development and adult function [45,46]. Consequently, availability of pollen is tightly linked to the number of honey bees that a colony can rear [47–50]. Colonies routinely experience seasonal pollen shortages when colonies deplete stores before more pollen can be collected [51–53], as often occurs during brief periods of bad weather [49,54–56] or as an unwanted byproduct of commercial management practices that put colonies in intense competition for pollen sources that may lack diversity, be poorly nourishing, or flower infrequently [57–63]. In response to pollen shortages, colonies tend to adjust the number of larvae they can rear through utilization of their own body reserves to support brood rearing [64] and by cannibalizing young larvae and investing reclaimed nutrients in older larvae [49,56,65]. Despite these protective measures, undersized and nutrient-depleted adults are sometimes reared when access to pollen is limited [66–68].

Previous studies that have tested the effects of manipulating larval nutrition on adults have utilized artificial diets, hand-reared larvae, caged adults, or some combination thereof (e.g., [69–75], but see [76] for an exception). However, the most realistic assessment of the consequences of worker undernourishment requires examining effects in a natural social context. Our study, which we repeated three times, meets this criterion. Because our focal workers lived in a normal social milieu, we were able to examine the effects of being reared in pollen-stressed colonies on two sophisticated tasks that are performed later in life by adult workers—foraging and recruitment. We created cohorts of nutritionally stressed and unstressed workers who were reared in colonies by nestmate nurses with access to either limited or adequate pollen stores. Focal workers were weighed at adult emergence and then co-fostered as adults in the same host colonies, where we monitored their longevity, foraging activity, and waggle-dance behavior. We found that pollen stress during larval development had far-reaching negative effects on task performance by adults later in life. Critically, performance deficits extended to foraging and recruitment, which are the most important tasks that honey bees perform as provisioners for their colonies and as pollinators of human-cultivated crops.

## Materials and Methods

### Manipulating pollen availability during larval development

In three separate trials conducted over two years, we compared the weight, longevity, foraging activity, and waggle-dance performance of honey bee workers that were reared as larvae under conditions of either limited or abundant pollen supply, but shared a common social environment as adults (2012: trial 1; 2013: trials 2 and 3; dance performance was examined in trial 3 only).

To manipulate developmental conditions for focal workers in each trial, we split source colonies into three subunits per colony, with the goal of creating one pollen-limited subunit that would exhaust its pollen supply during the development of focal larvae and another two subunits that were abundantly supplied with pollen so that focal larvae would be adequately provisioned during brood rearing (each was a different type of control; see below). A colony’s subunits were either left in the original hive or transferred to one of two 5-frame hive boxes. All colony subunits had larvae to rear, but they varied in the amount of pollen that they had to do so. One five-frame subunit was pollen limited: it had 1–2 frames of brood that contained <50 cm^2^ of pollen-filled comb in total; additional pollen was scraped out of frames and all remaining frames contained honey only (earlier tests of our method showed that colonies with more pollen did not reliably deplete their stores during brood rearing). Pollen-limited subunits were also prevented from collecting more pollen, as would occur during a period of unfavorable weather, by screening their entrances and placing them in a cool incubator for the duration of the development of the focal larvae (10–12°C; VWR low-temperature incubator, Radnor, PA, U.S.A.). We are confident that these colony subunits were pollen limited because their pollen stores were always gone by the end of the focal workers’ larval development, in contrast to the abundantly supplied controls (see below). Furthermore, out of the 23 source colonies that we subdivided over all trials, pollen-limited subunits from only 7 colonies produced enough brood to be included in the study because the remaining colonies cannibalized their brood, presumably in response to the pollen shortages that our manipulation created [49,56,66] (trials 1 and 3 used three source colonies each, trial 2 used one source colony). A second set of five-frame colony subunits matching these pollen-limited subunits was assembled from the source colonies and similarly confined, except that one of the food frames in each was at least 90% filled with pollen on one side (>775 cm^2^ of pollen-filled comb, faced toward the brood), in addition to pollen on any of the other frames (thus, confined controls with abundant pollen). The third set of subunits (the remainder of each source colony in the original hive) was given a similarly well-stocked pollen frame and was left unconfined so workers could collect more pollen from the environment (thus, unconfined controls with abundant pollen). Because there was always pollen left in control subunits at the end of the focal workers’ larval development (>50% of their original pollen frame), we are confident that the nurses who reared the focal workers had adequate access to pollen during larval provisioning. The colony subunits’ brood frames contained brood of all ages, but brood frames were carefully distributed among subunits so that each had a frame with eggs and/or young larvae (<2 days old). We did not tag emerging adults until at least 17 days after subunits were assembled, which ensured that focal workers experienced treatment conditions for most, if not all, of their larval development. Each source colony’s queen was left in the original hive subunit; the new hives received two lures impregnated with compounds that are produced by queens to signal their presence, which effectively suppress queen rearing [77] (lures were replaced every 2 days; colonies that received lures did not show signs of worker laying or queen replacement; Bee Boost, Phero Tech, Victoria, BC, Canada). Each colony subunit had 3–5 worker-covered frames shaken into them, depending on the size of the original source colony. In trial 1, there were no confined control workers and queen lures were not used; we report this trial nonetheless because its findings mirror those of the other two trials for which all control treatments were in place.

After focal larvae had been fed by their nestmates and sealed into cells to pupate, adult workers were removed from all brood frames, which were then transferred to a warm incubator (35°C) where workers completed their development. Frames were checked daily and, once focal adults began to emerge from sealed cells, they were individually weighed on an analytical balance (to the nearest 0.0001 g; AB104-S, Mettler Toledo, Columbus, OH, U.S.A) and then tagged on their thoraxes with uniquely identifiable numbered plastic discs (BioQuip, CA, U.S.A. and Chr. Graze, Weinstadt, Germany) before introduction into their trial’s observation hive on the same day of emergence. Focal workers emerged over a 7-day period in trial 1 and a 4-day period in trials 2 and 3; across trials, mean number of focal workers introduced per source colony was similar among treatments (122 ± 30 workers per pollen-limited colony subunit; 121 ± 24 workers per confined control with abundant pollen; 128 ± 18 workers per unconfined control with abundant pollen; F_2,15_ = 0.2, *P* = 0.98). Observers were not aware of the treatment to which workers belonged during subsequent data collection. For each trial, a host colony was installed in a queenright, two-frame observation hive, which was set up indoors in a hive house in the Wellesley College Arboretum (Wellesley, MA, U.S.A.). All source and host colonies (and their queens, who were of mixed descent and less than one year old) were maintained prior to each trial in the Wellesley College research apiaries after being purchased from local bee suppliers (2012: Beehavin’ Apiaries, Smithfield, RI, U.S.A.; 2013: Merrimack Valley Apiaries, Billerica, MA, U.S.A.). Workers in each observation hive could forage outdoors by walking through a tube that connected the hive to an opening in a wall of the building. All trials were conducted during the summer months (June–August) and host colonies maintained honey and pollen stores for the duration of that period (the host colony in trial 3 ran low on food at one point, so one of its frames was replaced with a frame containing pollen and honey). All source and host colonies were thriving and appeared disease free prior to the start of each trial.

### Assessing the longevity and foraging performance of focal workers

The longevity of focal workers was determined by checking observation hives two times per day after the first tagged workers were introduced into them until no more workers were found. We conducted most checks in the early morning and evening, when foraging activity was minimal and we had the greatest chance of seeing all tagged workers. Records of foraging activity (see below) were combined with hive-check data to improve the accuracy of longevity estimates. We noted the identities of tagged workers who were not seen in their observation hive 24 hours after their introduction to it; only those workers who were present for longer than 24 hours were included in comparisons of treatment means for worker weight and performance.

Foraging activity was assessed by monitoring each hive’s entrance for focal workers over a 2-hour period between 9 AM and 5 PM each day. A plexiglass-covered runway (26 x 10 x 3 cm) was attached to each entrance to facilitate the observation of tagged foragers. A series of staggered baffles increased the distance that entering and exiting bees had to travel, which slowed most workers enough to permit their identification. Observations of foraging began when frequent checks revealed that focal workers were present at their hive entrance (day 7 in trials 1 and 2 and day 6 in trial 3) and ended when the number of focal workers had declined to the point that they were rarely observed (day 50 for trial 1; day 26 for trial 2; day 52 for trial 3). Participation in foraging, age at onset of foraging, and number of days observed foraging were compared among treatment groups for each trial.

### Assessing the waggle-dance performance of focal workers

The effect of being reared in pollen-limited colonies during larval development on adult waggle-dance behavior was estimated for focal workers in trial 3. Each observation hive had a single “dance floor’—the area of comb adjacent to the entrance where foragers dance—because a shunt forced all entering foragers to come in on the same side of the bottom frame. The dance floor was videotaped for 1–2 hours per day between 9 AM and 5 PM as weather and forager activity permitted (using diffuse overhead fluorescent lighting), starting when focal workers were first observed dancing during frequent checks of the dance floor (when the youngest workers were 12 days old) and ending when only one dance was performed per hour during the final day of taping (when the oldest workers were 45 days old; 41 hours of video in total; Sony HandyCam DCR-HC63, Tokyo, Japan). Focal foragers performing waggle dances were identified on each videotape by an observer, which were later analyzed frame-by-frame (one frame = 1/30 s) using Final Cut Express 4.0.1 (Apple Inc., Cupertino, CA, U.S.A.) to estimate dance metrics. A single waggle dance included all waggle runs that were performed by a forager upon return to the hive before she either left to forage again or moved deeper into the hive. A single dance by a forager often consists of multiple bouts of dancing broken up by pauses to change location or to transfer food to other workers or into cells; total waggle runs were summed for all segments of a dance that were interrupted by such pauses. Several aspects of waggle-dance activity were compared among treatment groups for dances performed by focal workers as they foraged freely at unknown food sources: participation in dancing, number of days observed dancing per dancer, total dances per dancer, total number of waggle runs per dancer, and average runs per dance for each dancer. Waggle-run duration (as a proxy for distance to food sources) was also compared among a subset of workers to determine whether developmental pollen stress affected the distance at which workers foraged, as least according to the locations that were advertised by dances. To do this, mean waggle run duration was estimated for the first dance that each pollen-limited dancer performed, which was compared to the mean run duration for the first dances performed by workers from both controls who danced during the same hours of video.

In addition to naturally available forage, a small number of focal workers visited a sucrose-solution feeder that was set up to examine the precision of the waggle runs performed by workers as they recruited to a known food source at a fixed location. Dance precision was assessed as variability in the angle and duration of a dance’s waggle runs—its direction and distance components, respectively—for dances performed by focal workers after they returned from the feeder (measured as the standard deviation of mean run angle and duration for each dance, which were then compared among treatments). To ensure that enough workers from each treatment visited and danced for the feeder, we introduced extra workers into the observation hive from each colony/treatment combination in trial 3. The number of unique tags was limited, so these extra workers were marked with a paint color that designated their origin (source colony and treatment). Thus, variability in dance angle was estimated for paint-marked and individually tagged workers who visited the feeder, whereas all other analyses of waggle-dance behavior (above) were estimated for tagged workers only. Because paint-marked could not be distinguished as individuals, dances for the feeder were viewed as independent records. The feeder was set up 188 m north of the observation hive and stocked with 1.5 M or 2.0 M anise-scented sucrose solution (depending on forager interest). Unmarked bees were removed from the feeder and caged until the feeder was emptied to limit visitation to focal workers only. An observer at the feeder relayed the identities of visiting focal workers to the observer who was videotaping the dance floor and pointing out their dances (which were also distinguishable because they were generally similar). Workers who danced for the feeder visited it between days 19 and 29 of trial 3.

### Statistical approach

Numbers of focal workers that did or did not participate in a task were compared among treatments with 2x2 (trial 1) or 2x3 contingency tables (trials 2 and 3) using chi-square tests of independence. We expected that differences in mean worker weight and performance would exist among colonies and across trials (which were conducted during different years and months of the summer). However, our focus was not on these temporal and genetic influences on worker metrics, but rather on whether the effects of colony-level pollen stress were expressed consistently in worker performance in each trial. Therefore, we compared treatment means separately for each trial by t-test (trial 1) or ANOVA (trials 2 and 3) and applied a Bonferroni correction to α = 0.05 to account for conducting multiple tests. Post-hoc pairwise comparisons were made using the Tukey-Kramer procedure where treatment effects were significant. The survival of workers reared in pollen-limited and pollen-abundant treatment conditions was compared with Kaplan-Meier estimates of survival; post-hoc comparisons were made with Šidák adjustments to log-rank tests. T-tests, ANOVAs, and survivorship analyses were conducted using SAS version 9.3 (SAS Institute, Inc., Cary, NC, U.S.A.); contingency tests were conducted with an open-access statistical calculator (physics.csbsju.edu/stats/contingency.html). The dataset is available as Supporting Information (S1 File).

## Results

### Adult weights were lowest when larvae were reared in pollen-limited colonies

Across the three trials, a total of 1,808 workers were successfully introduced into observation hives (i.e., they were present for at least 24 hours after introduction to their observation hive: 638 workers reared in pollen-limited, confined colonies, 410 workers reared in confined colonies with abundant pollen, and 760 workers reared in unconfined colonies with abundant pollen). Analyses for each trial considered accepted focal workers only.

**Fig 1:**
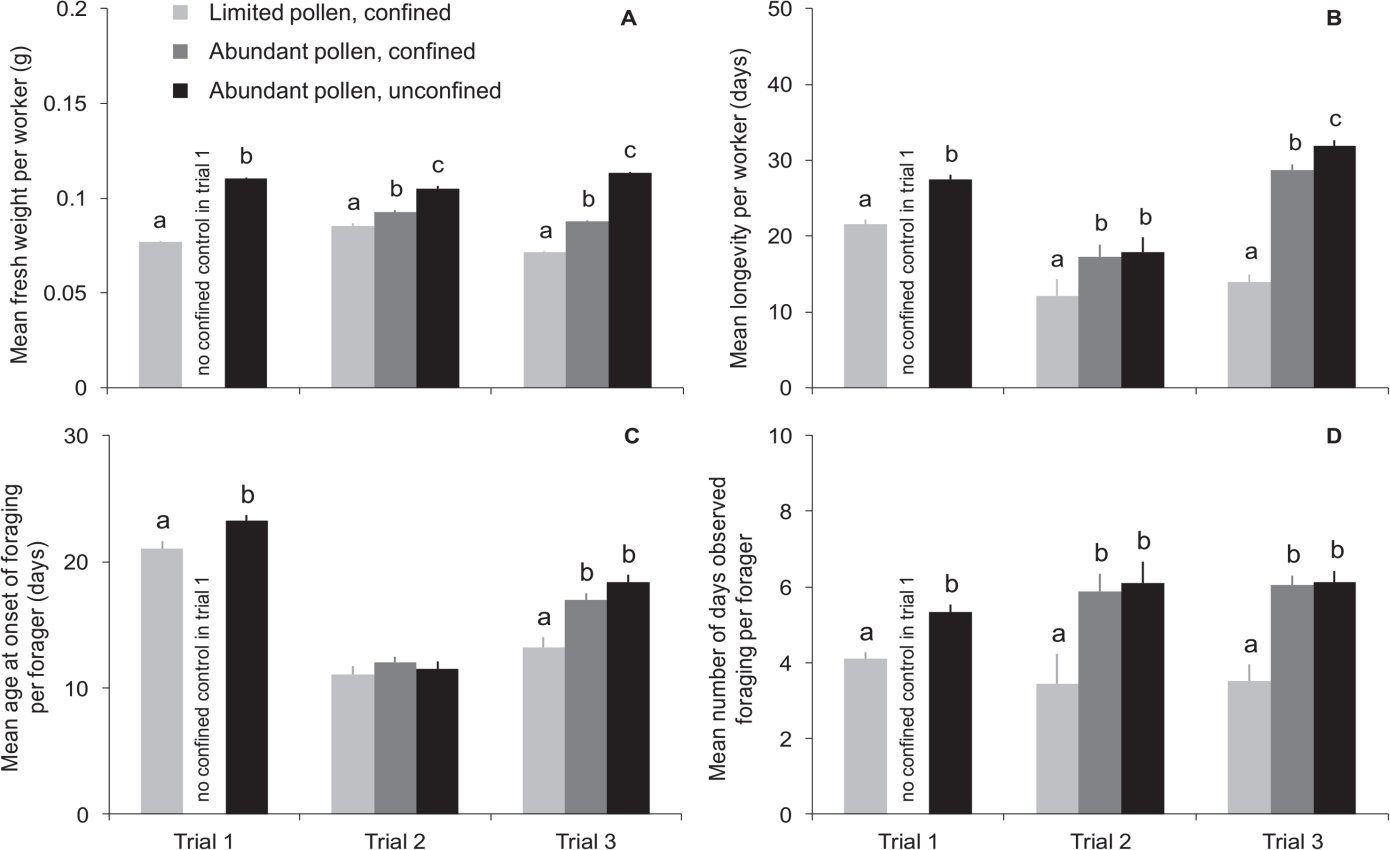
Weight, longevity, and foraging activity of adult workers were reduced when access to pollen was limited during larval development. Mean (± SEM) A) fresh weight of focal workers at adult emergence, B) longevity of focal workers, C) age at onset of foraging for focal workers who foraged, and D) number of days observed foraging for focal workers who foraged. Focal workers originated from source colonies that were split into colony subunits that had either limited or abundant supplies of pollen when focal workers were reared as larvae. Subunits were either confined to a cool incubator to prevent further pollen foraging (pollen-limited or confined controls) or allowed to forage freely (unconfined controls). When development was complete, focal workers were co-fostered as adults in an unrelated host colony. The experiment was replicated in three separate trials that used different source and host colonies. Means comparisons were made within trials wherever treatment effects were significant after a Bonferroni correction; significant differences between treatments are indicated by letters.

Focal workers reared under conditions of pollen limitation had reduced weight compared to workers that were reared in the confined and unconfined controls (Fig. 1A; trial 1: t_914_ = 52.6, *P* < 0.0001; trial 2: F_2,143_ = 56.2, *P* < 0.0001; trial 3: F_2,737_ = 1336.5, *P* < 0.0001). Across trials, workers that experienced pollen limitation as larvae were 8–37% lighter at adult emergence than workers that were reared by nestmates with access to abundant pollen (controls). Significant differences between control treatments in mean emergence weight indicated an effect of being reared by workers who were confined to the hive, even with plentiful supplies of pollen (Fig. 1A).

Across the three trials, more workers from pollen-limited colonies were unsuccessfully introduced into their observation hive (i.e., not seen again after 24 hours) than workers reared in the confined and unconfined control colonies (26% versus 16% and 12%, respectively; all trials pooled: χ^2^ = 58.2, df = 2, *P* < 0.0001). This difference was consistent within trials (trial 1: χ^2^ = 48.9, df = 1, *P* < 0.0001; trial 2: χ^2^ = 26.6, df = 2, *P* < 0.0001; trial 3: χ^2^ = 35.8, df = 2, *P* < 0.0001).

### Adult lifespan was shortest when larvae were reared in pollen-limited colonies

Adult longevity was substantially and consistently lowered when focal workers were reared as larvae in colonies that were pollen limited compared to workers reared in colonies that had abundant pollen (Fig. 1B; trial 1: t_919_ = 7.1, *P* < 0.0001; trial 2: F_2,143_ = 6.7, *P* = 0.0016; trial 3: F_2,737_ = 80.7, *P* < 0.0001). Mean lifespan was reduced at minimum by 5 days and at most by 18 days for pollen-limited workers compared to workers in control groups (Fig. 1B), which represents a decrease in mean longevity of 21–56% across trials.

**Fig 2:**
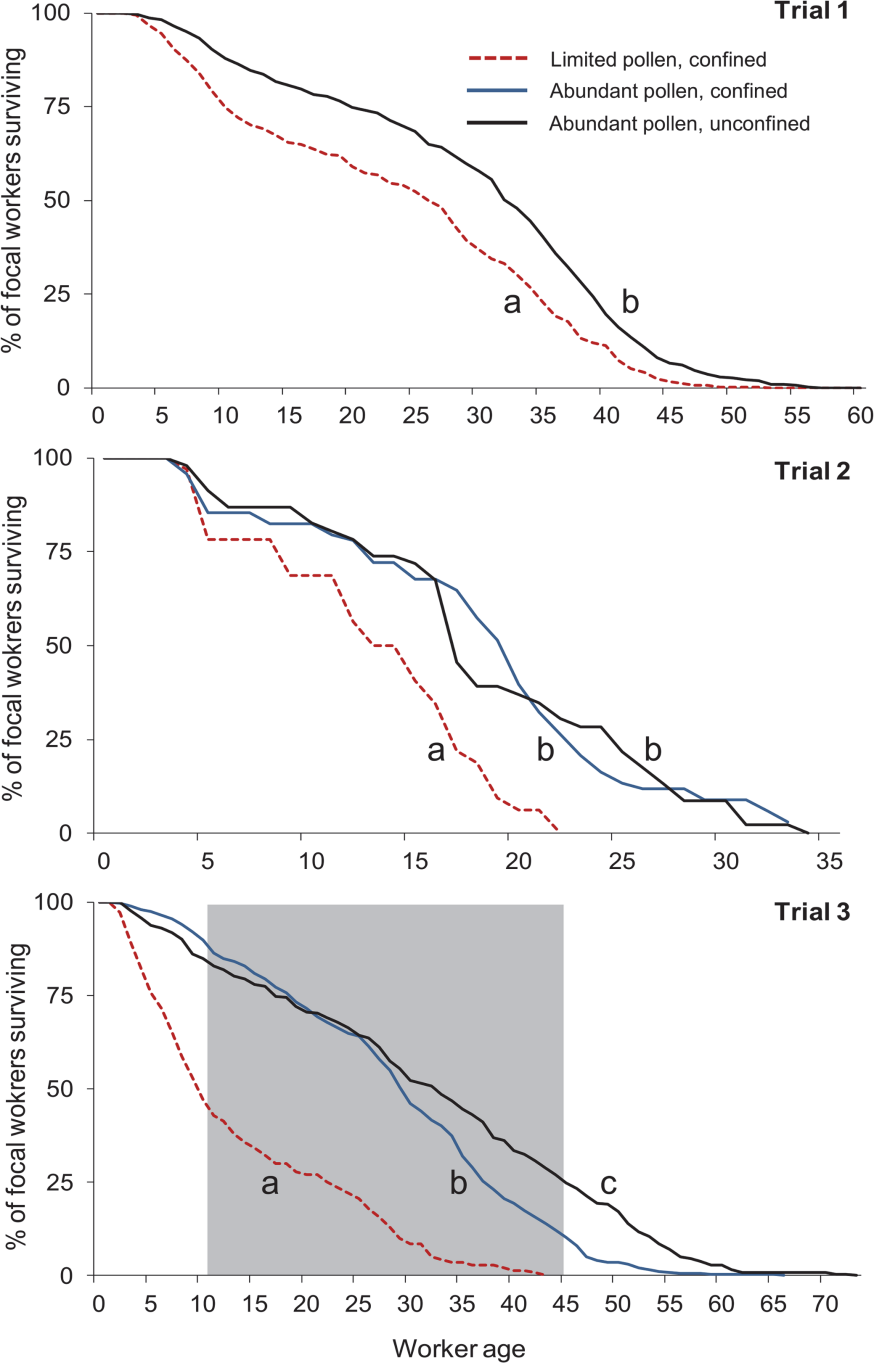
Survivorship of adult workers was lowest when access to pollen was limited during larval development. Workers were either reared in colonies with limited pollen (and confined to prevent further foraging) or reared in colonies with abundant pollen (either confined or allowed to continue to forage; controls). In each trial, focal workers were introduced into an observation hive after adult emergence; only those workers present 24 hours later were included in the survivorship analysis. The gray area in trial 3 indicates the period over which waggle-dance recruitment was monitored. Raw data are depicted, pooled across colonies within a treatment per trial, rather than Kaplan-Meier estimates of survival function (see Methods). Significant differences in survival among treatments within a trial are indicated by different letters.

There was no difference in mean worker longevity between control treatments in trial 2 (Fig. 1B). In trial 3, mean worker longevity was 3 days shorter for focal workers reared in confined control colonies compared to workers reared in unconfined controls (Fig. 1B). However, this difference was small compared to the reduction in longevity experienced by workers reared in pollen-limited colonies in the same trial—they lived 18 fewer days on average compared to workers from unconfined controls and 15 fewer days than workers from confined controls (Fig. 1B). The survivorship of workers reared in pollen-limited colonies was significantly lower than that of workers from both control treatments in each trial (Fig. 2; log-rank tests of survival function with Šidák adjustments; *P* < 0.0001 for each trial).

### Foraging activity was reduced when reared in pollen-limited colonies

Across all three trials, availability of pollen during larval rearing significantly affected the percentage of workers who were observed foraging as adults (χ^2^ = 65.6, df = 2, *P* < 0.0001). Only 62% of workers who were reared in pollen-limited colonies were observed foraging at some point during their lifetime compared to 81% and 80% of workers reared in the abundantly supplied controls (confined and unconfined treatments, respectively). When examined separately, this result was consistent in only two of three trials after a Bonferroni correction (trial 1: χ^2^ = 40.8, df = 1, *P* < 0.0001; trial 2: χ^2^ = 6.4, df = 2, *P* = 0.04; trial 3: χ^2^ = 14.2, df = 2, *P* = 0.0008).

Of the focal workers who foraged, those reared in pollen-limited colonies tended as adults to begin foraging earlier and consistently foraged for fewer days compared to their control counterparts. The onset of foraging was accelerated by 2 days on average for foragers from pollen-limited colonies in trial 1 (t_657_ = 3.0, *P* = 0.003; Fig. 1C) and by 4–5 days on average in trial 3 (F_2,563_ = 13.4, *P* < 0.0001; Fig. 1C). Foraging was initiated at a similar age across all treatments in trial 2 (F_2,109_ = 0.7, *P* = 0.51; Fig. 1C). Once foraging, workers reared in pollen-limited colonies were observed foraging on fewer days than workers from control treatments: 1 day less on average in trial 1 (t_657_ = 4.7, *P* < 0.0001; Fig. 1D), 2–3 days less in trial 2 (F_2,109_ = 4.3, *P* = 0.016; Fig. 1D), and 3 days less in trial 3 (F_2,563_ = 13.8, *P* < 0.0001; Fig. 1D).

Overall, foragers reared in pollen-limited colonies were far more likely to die (i.e., disappear from record) after being observed foraging for only a single day compared to foragers reared in the abundantly supplied controls (30% of foragers reared in pollen-limited colonies disappeared after one day versus 15% and 13% of foragers from confined and unconfined control treatments, respectively; χ^2^ = 51.6, df = 2, *P* < 0.0001). This effect was strong in two of three trials when examined separately (trial 1: χ^2^ = 28.9, df = 1, *P* < 0.0001; trial 2: χ^2^ = 3.1, df = 2, *P* = 0.21; trial 3: χ^2^ = 32.4, df = 2, *P* < 0.0001).

### Dance activity and precision were reduced when reared in pollen-limited colonies

A total of 397 dances were performed by 116 uniquely tagged focal workers as they visited a variety of food sources during the 32-day period that the dance floor was monitored in trial 3 (n = 9 dancers reared in pollen-limited, confined colonies; n = 66 dancers reared in confined colonies with abundant pollen; n = 41 dancers reared in unconfined colonies with abundant pollen). Of the focal workers who were observed foraging, only 9% of workers reared in pollen-limited colonies were also observed dancing compared to 24% and 21% of workers reared in the abundantly supplied confined and unconfined control colonies (χ^2^ = 9.8, df = 2, *P* = 0.008; or 6% versus 19% and 16% of workers that were successfully introduced to the hive, respectively: χ^2^ = 14.2, df = 2, *P* = 0.001). We began videotaping the dance floor as soon as we observed focal individuals dancing during frequent hive checks. However, the percentage of workers who danced may be underestimated across treatments because any dances performed by foragers who died before filming began were not recorded, and this omission may disproportionately affect shorter-lived workers who were reared in pollen-limited colonies (Figs. 1B, 2). If we consider the pool of potential dancers to be only the workers who foraged during the period that the dance floor was videotaped, then dance participation was still lowest for workers reared in pollen-limited colonies, but not significantly so (14% of pollen-limited workers versus 28% and 23% of workers from the confined and unconfined controls; χ^2^ = 4.6, df = 2, *P* = 0.10). Counterbalancing this argument is the fact that foragers reared in pollen-limited colonies were observed dancing less often and far more of them died after being observed foraging on only a single day (see previous section), so these foragers had a lower likelihood of contributing to recruitment in the first place.

**Fig 3:**
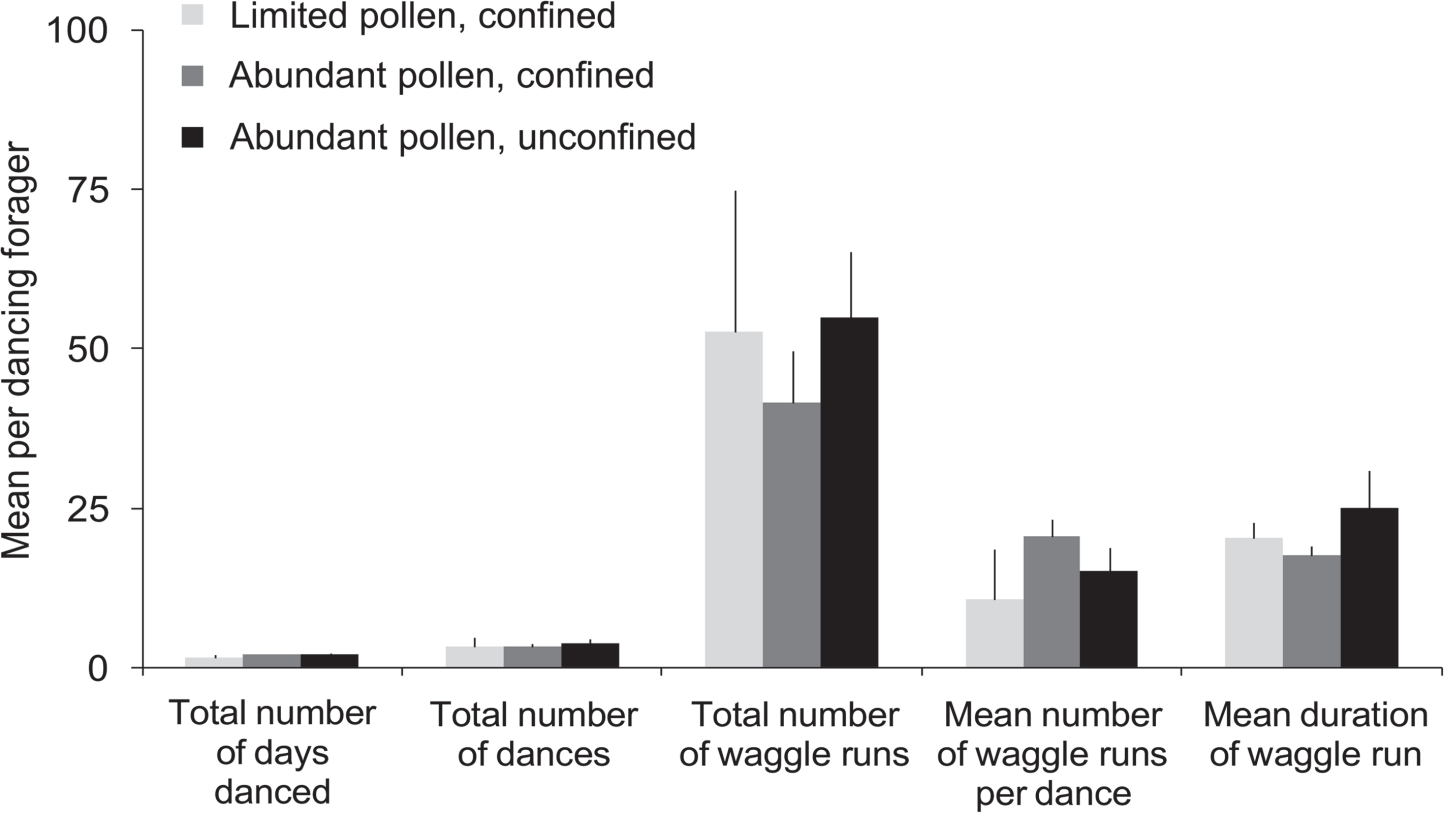
Waggle-dance behavior of adult workers was not affected by access to pollen when focal workers were larvae. Provided are mean per dancer measures of dance performance (± SEM) as focal individuals foraged at unknown food sources in trial 3. All workers were uniquely tagged and individually identifiable. Waggle-dance activity was monitored for 1–2 h/day (as weather and foraging permitted) from the time that workers were min. 12 days to max. 45 days of age (see gray box in Fig. 2). Means were calculated considering only those workers who danced (i.e., zero values were not included for non-foraging or non-dancing focal workers; n = 9 workers reared in pollen-limited, confined colonies; n = 66 workers reared in abundantly supplied, confined controls; n = 41 workers reared in abundantly supplied, unconfined controls). Mean waggle-run duration (number of frames at 30 frames per second, a proxy for distance to advertised food source) was estimated for the first dance performed by each pollen-limited worker and compared to means for the first dances performed by control workers during the same hours of videotape (n = 9 workers reared in pollen-limited, confined colony subunits; n = 20 workers reared in abundantly supplied, confined controls; n = 13 workers reared in abundantly supplied, unconfined controls).

**Fig 4:**
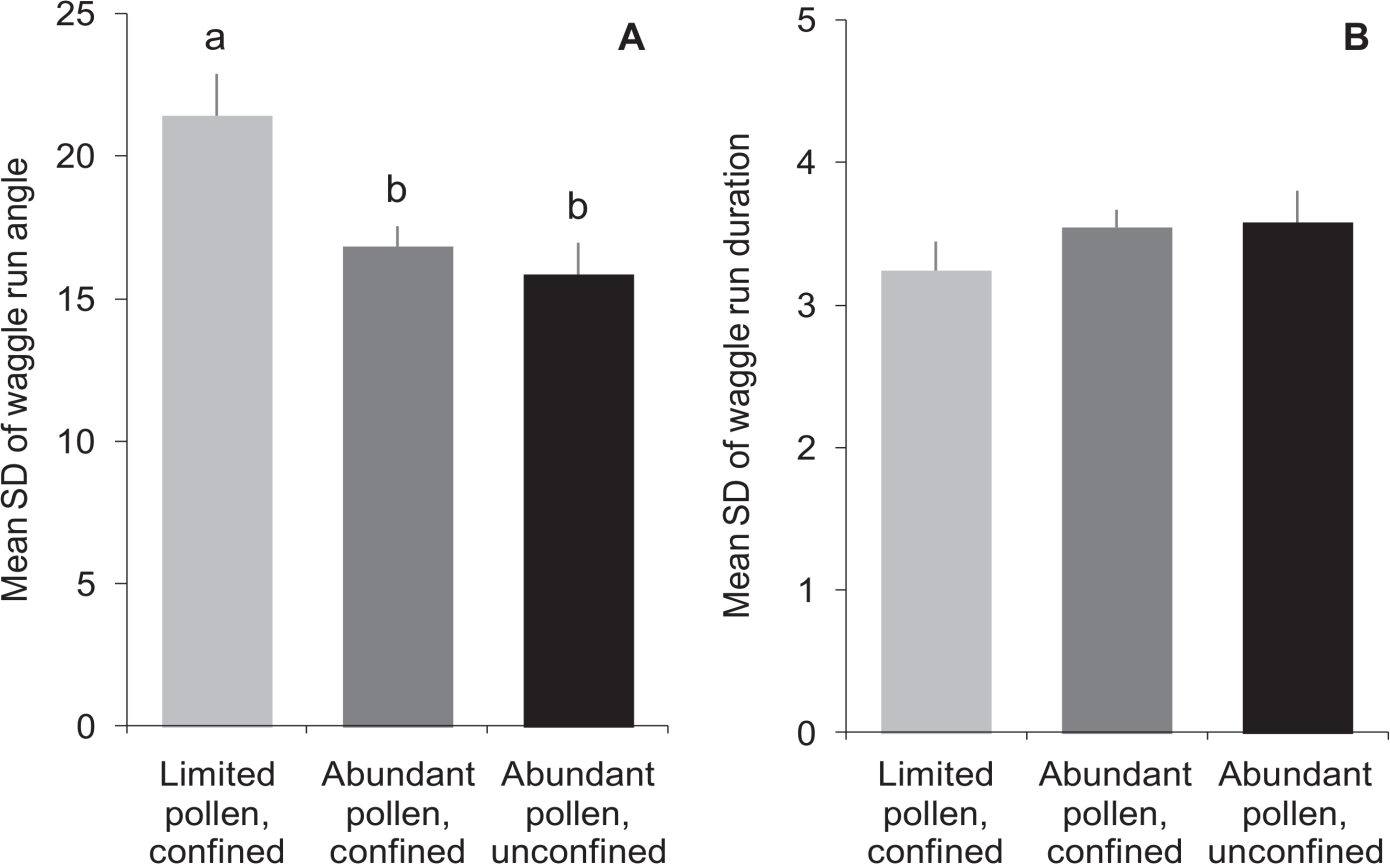
Workers reared in colonies with limited pollen performed waggle dances with greater directional imprecision as adults. Variability in the A) direction and B) distance components of waggle dances were estimated for workers who were either reared in colonies with limited pollen (and confined to prevent further foraging) or reared in colonies with abundant pollen (either confined or allowed to continue foraging; controls). All dances were performed for a sucrose-solution feeder that was maintained at a fixed location from the observation hive in trial 3. Feeder dances were performed by workers who had treatment-specific marks (paint marks and tags), but individuals were not always uniquely identifiable, so each dance was treated as an independent record. Standard deviations (SD) of the angle and the duration of the waggle runs for each dance were calculated to compare directional precision among treatments. Differences between treatments are indicated by letters where significant treatment effects were found.

Once engaged in dancing, the level of pollen stress that workers experienced during development did not affect the amount of dancing that they did, but it did affect the precision of their dances. As tagged workers foraged at unknown food sources, the mean total number of days that each focal dancer danced, the total number of dances observed per dancer over those days, the total waggle runs performed, mean number of waggle runs per dance, and mean waggle-run duration (a proxy for distance to advertised food sources) did not differ across treatments (Fig. 3; F_2,113_ = 0.3, *P* = 0.72; F_2,113_ = 0.1, *P* = 0.88; F_2,113_ = 0.6, *P* = 0.58; F_2,113_ = 1.1, *P* = 0.33; F_2,39_ = 1.2, *P* = 0.32; respectively). Similarly, paint-marked and tagged workers from all treatments performed similar numbers of waggle runs upon return from the sucrose-solution feeder (Fig. 4; based on the number of painted and tagged workers: 27 dances performed by at least 6 different workers reared in pollen-limited colonies, 63 dances by at least 18 different workers from confined control colonies, and 35 dances by at least 9 different workers from unconfined control colonies; F_2,122_ = 2.2, *P* = 0.11). However, feeder dances performed by pollen-limited workers conveyed more variable information about the direction of the feeder (i.e., angles of waggle runs in each dance) than dances performed by control workers (Fig. 4; F_2,122_ = 5.7, *P* = 0.002). Variability in the distance component of the dances (i.e., durations of waggle runs in each dance) was similar among treatments (Fig. 4; F_2,122_ = 0.4, *P* = 0.88).

## Discussion

The foraging and recruitment performance of workers as adults was substantially compromised if they were reared as larvae in a pollen-limited colony environment. Compared to nestmates who were reared under conditions of pollen abundance, pollen-stressed workers were lighter, they died sooner, and fewer of them were observed foraging. Those who did forage initiated foraging sooner, foraged for fewer days, and were more likely to die after a single day of foraging. In addition to being less likely to forage, workers reared in pollen-limited colonies were also less likely to waggle dance than control workers and, if they danced, their dances were less precise (although they danced with similar effort). These effects suggest a lasting legacy for workers of nutritional stress, one that seriously compromises the foraging and recruitment ability of adults, even when stress is restricted to the larval stage only. It is likely that these behavioral deficits would be exacerbated if chronic stress persisted throughout adulthood at the worker level, and if a greater proportion of workers were undernourished at the colony level. These effects may also escalate over time if stressed foragers cannot collect enough food to adequately provision larvae, who may then become underperforming adults themselves. Given the central role that foraging and recruitment productivity play in colony survival [78–80] and the utility of honey bees as crop pollinators [27], these scenarios, supported by our findings, justify concerns about the role of poor nutrition in colony decline.

Foraging and recruitment are the final tasks that honey bees perform during their lifetime, so it may not be surprising that the cumulative effects of developmental pollen stress are strongly manifested in this end-point suite of behaviors, and in nuanced ways. Similar effects on foraging and recruitment have been induced by other environmental stressors throughout workers’ lives. Sleep deprivation [81] and low developmental temperature [82] reduce the precision of waggle dances and, as in this study, these effects are sometimes apparent in only one element of the dance (i.e., distance versus dance components) [82]. Low developmental temperature [82] and exposure to pesticides [83] can also reduce the probability and extent to which workers waggle dance after foraging. While it is remains possible that the probability of dancing for pollen-stressed workers was underestimated in our study, it is likely that it was not. No dances by focal workers were observed prior to the start of videotaping in trial 3, despite repeated checks each day to determine when taping should begin. Moreover, workers reared in pollen-limited colonies were more likely than workers from adequately provisioned colonies to disappear after their first day of foraging, suggesting that they may have had difficulty returning to their hive, possibly because of an inferior ability to evade predation, insufficient vigor, poor homing ability, or another physical limitation brought on by undernourishment. Such forager losses and inability to home are also found in workers that are afflicted by other environmental stressors (e.g., pests, pathogens, pesticides, and viruses [84–88]). Greater likelihood of disappearing after one day of foraging means that proportionally fewer nutritionally stressed workers would be expected to dance, as we found. Finally, homing ability and foraging efficiency are highly dependent on worker learning [89], which may be impaired in undernourished workers if such developmental stress disrupts adult learning and memory, as it does in vertebrates [8,90–93] and in fruit flies [25]. Other environmental stressors impair honey bee learning, including pests and pathogens [94,95], exposure to pesticides [83,96], and temperature stress [82,97], suggesting the possibility of a similar effect with nutritional stress.

Our findings confirm in a natural colony setting the consistent finding that developmental pollen stress reduces adult weight and longevity when workers live outside of a social context (i.e., in cages) [71,73–75]. Studies of the effects of larval pollen stress on workers reared and living in colonies are uncommon and tend not to focus on worker behavior [69], with the exception of the finding that larval pollen stress hastens the onset of foraging [76] (which we confirmed). Early onset of foraging for developmentally stressed individuals may be linked to relatively faster depletion of nutritional reserves, which precedes the switch from indoor to outdoor tasks in honey bees [98] and other social insects [99,100]. Adequate access to pollen can increase the duration of indoor nursing activity [101], thus delaying the onset of foraging, but pollen availability was equivalent for co-fostered adults in our study, which means that the behavioral transition to outdoor tasks was accelerated in part by the poor nutritional status of workers that were pollen stressed as larvae (likely truncating nursing activity as well). Furthermore, it suggests that the behavioral effects of developmental nutritional stress cannot be fully rescued by regular access to food later in life.

Honey bee larvae are probably routinely exposed to the short-term nutritional stress that was experienced by our focal individuals, either seasonally or because of management practices that limit nutrient availability. This is suggested by the overlap between the weight range for pollen-stressed and unstressed workers in this study (mean 71–113 mg across treatments and trials) and weights reported previously for workers at adult emergence (81–140 mg; reviewed by [102]). Larvae undergo a 700-fold weight gain during the 5–6 days that they are nursed [103], but a single day of bad weather reduces nursing activity by more than one half, even when colonies have stored pollen [104]. This response to poor weather likely explains in our study why confinement alone (without pollen stress) produced workers that were slightly smaller than those reared in unconfined colonies (but with few behavioral effects). Over the long term, the number of small workers in colonies increases with repeated bouts of bad weather [54] and the heaviest workers are reared at times when pollen is readily available within a season [68], so differences among our treatments in emergence weights likely reflect adjustments made to brood provisioning in colonies in response to changes in both foraging opportunity *and* pollen stores (but note that confinement had no effect on the behavior of abundantly supplied control workers). Because the window during which larvae are fed is so brief, day-to-day changes in attention from nurses have the potential to generate nutritional stress for developing workers and the corresponding deficits in adult function that we demonstrated here. It is worth noting that pollen-limited workers often looked similar in size to control workers, so it would be difficult to determine by visual inspection alone that workers in managed colonies had been subjected to such stress.

A chief concern about the impact of poor nutrition on honey bee colonies is the possibility that it acts synergistically with other environmental stressors to undermine colony function. Notably, undernourished larvae are particularly vulnerable to some of these stressors, including pests, pathogens, and pesticides. High levels of pesticides in brood comb during larval development reduce adult longevity [105], which would likely be compounded by larval pollen stress because pesticides are more toxic to protein-deficient workers [106]. Furthermore, pesticide exposure of cells in the midgut, the site of nutrient adsorption, increases cell death in both adult and larval workers [107,108], but the effects are especially pronounced for larvae. Susceptibility to economically damaging brood pathogens is also worsened when larvae are undernourished [72,109,110]. Conversely, pupae parasitized with *Varroa destructor* mites partly overcome the physiological and behavioral symptoms of infestation if they are reared as larvae with plentiful access to pollen [76], but symptoms are not mitigated if adequate nourishment is delivered to workers only when they are adults [43]. Finally, adults infected with the gut parasite *Nosema* exhibit many of the same symptoms that we generated with larval pollen limitation alone (reduced longevity, early onset of foraging; [111,112]), so these two stressors may act synergistically, even though exposure occurs during different stages of life. Unfortunately, the symptoms of larval pollen stress reported here mimic the negative effects on workers of these environmental stressors that also impair workers’ access to nutrients [107,108,113–115]. Together, multiple stressors may coalesce into a perfect storm of conditions that make it difficult for workers to acquire the nourishment that they need to function as efficient foragers and dancers. This possibility, which is suggested by our findings, warrants concern about the effect of poor nutrition on the health and productivity of honey bee colonies and the quality of pollination services that they can offer.

## Supporting Information

S1 File Dataset for Figs. 1–4 and contingency table calculations. (XLSX)
